# Unveiling the Therapeutic Potentials of Mushroom Bioactive Compounds in Alzheimer’s Disease

**DOI:** 10.3390/foods12152972

**Published:** 2023-08-07

**Authors:** Na Li, Hongbo Li, Zhenbin Liu, Gao Feng, Chunyang Shi, Yue Wu

**Affiliations:** 1 College of Food Science and Engineering, Central South University of Forestry and Technology, Changsha 410004, China; 20200100082@csuft.edu.cn; 2 School of Food Science and Engineering, Shaanxi University of Science and Technology, Xi’an 710021, China; hongbo715@163.com (H.L.); zhenbinliu@sust.edu.cn (Z.L.); 15339246223@163.com (G.F.); shichunyang@sust.edu.cn (C.S.)

**Keywords:** mushrooms, bioactive compounds, Alzheimer’s disease, neuroprotection

## Abstract

Alzheimer’s disease (AD) stands as a prevailing neurodegenerative condition (NDs), leading to the gradual deterioration of brain cells and subsequent declines in memory, thinking, behavior, and emotion. Despite the intensive research efforts and advances, an effective curative treatment for the disease has not yet been found. Mushrooms, esteemed globally for their exquisite flavors and abundant nutritional benefits, also hold a wealth of health-promoting compounds that contribute to improving AD health. These compounds encompass polysaccharides, proteins, lipids, terpenoids, phenols, and various other bioactive substances. Particularly noteworthy are the potent neuroprotective small molecules found in mushrooms, such as ergothioneine, erinacine, flavonoids, alkaloids, ergosterol, and melanin, which warrant dedicated scrutiny for their therapeutic potential in combating AD. This review summarizes such positive effects of mushroom bioactive compounds on AD, with a hope to contribute to the development of functional foods as an early dietary intervention for this neurodegenerative disease.

## 1. Introduction

Alzheimer’s disease (AD) is a neurodegenerative disorder occurring frequently in aging people, characterized by a progressive loss of memory and cognitive functions. As the population grows and ages, the proportion of AD patients becomes larger, which will cause heavy health care, economic, and social burden. AD was first described by Alois Alzheimer in 1907 in a patient named Augustine Deter [1]. The main pathological characteristics of AD include the extracellular beta-amyloid (Aβ) plaques deposition and tau tangles (Tau). The main clinical manifestation of AD is progressive decline in memory power; some may also face personality and behavioral changes. Currently, the mainstream direction of research on the pathogenesis of AD is to focus on Aβ and Tau, but the pathogenesis of AD remains unclear [2]. Research indicated that the development of AD is related to a variety of factors, such as Aβ deposition and tau protein aggregation [3]. Various hypotheses, including cholinergic, inflammation, oxidative stress, mitochondrial dysfunction, and gut microbiome have also attracted much research and attention [4]. AD is considered to be a multifactorial disease associated with multiple risk factors, such as age, genetics, lifestyle, vascular disease, infection, and environmental factors [5]. Hence, the most effective way to prevent and treat AD is to use a multifaceted approach that includes appropriate nutrition, physical exercises, stimulating intellectual and social activities, and stress-reduction techniques. For patients who already have AD, treatment is geared toward managing symptoms and slowing the progression of the disorder.

**Figure 1:**
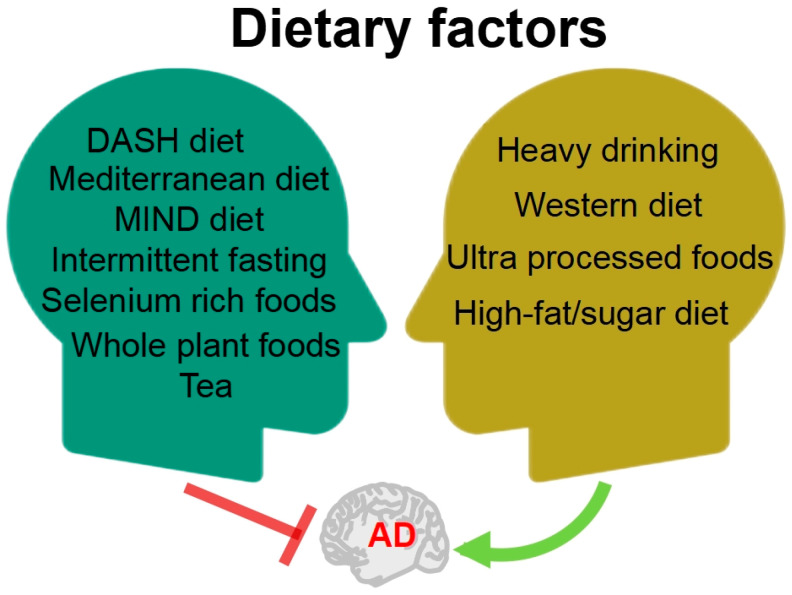
The relationship between diet factors and AD. DASH: dietary approaches to stop hypertension; MIND: Mediterranean-DASH intervention for neurodegenerative delay.

In recent years, numerous persuasive evidences suggested that dietary factors may be a critical factor in both the treatment and prevention of AD [6,7]. Studies have explored a range of specific foods and nutrients, dietary patterns, and other approaches that may have the potential to prevent or treat AD (Figure 1). Scientific evidence suggests that following the Mediterranean, DASH, and MIND diets is linked to lower cognitive decline and reduced risk of AD, with the MIND diet showing the strongest correlation [8]. Furthermore, evidence has shown that caloric restriction by intermittent fasting showed a wide range of beneficial effects on AD pathology from multiple perspectives [9]; years of intermittent fasting probably delayed or reversed the pathological process of AD. Selenium is an essential trace element in the human body; a review by Chen et al. [10] summarized that selenium-rich foods and their active ingredients resulted an improvement in AD through antioxidant, anti-inflammatory, and autophagic regulatory effects. Whole plant foods, such as mushrooms, berries, garlic, and turmeric, were found to effectively prevent and improve cognitive deficit via regulating the main pathway of neuroinflammation, lipoxin A4 (LXA4)-nuclear factor-kappa B (NF-κB), and mitogen-activated protein kinases (MAPK). These beneficial effects were mainly attributed to their high contents of functional macromolecules, including polysaccharides, bioactive peptides, and polyphenols; therefore, whole-plant foods can be part of a dietary plan to prevent the progression of AD [11]. Tea is the world’s most consumed beverage, originating from China. Huang et al. [12] summarized the effects of tea and its active compounds on the prevention and regulation of AD.

In parallel, poor dietary habits can exacerbate the condition of AD (Figure 1). A retrospective cohort study of 3,933,382 individuals in Korea showed that low alcohol consumption was associated with a reduced risk of dementia; mild to moderate alcohol consumption was associated with a decreased risk of dementia; whereas heavy drinking of alcohol was associated with an increased risk of dementia [13]. The dietary pattern known as the Western diet, which contains excessive amounts of saturated fatty acids and simple sugars, is one of the risk factors for AD. Based on the research data from both humans and experimental animals, the Western diet was found to evoke memory impairment by accelerating metabolic syndrome and systemic inflammation, causing damage to the blood–brain barrier (BBB) [14]. A large prospective study conducted by researchers from Tianjin Medical University, covering over 70,000 people and following up for 10 years, indicated a positive correlation between higher intake of ultra-processed foods, characterized by “high sugar, high fat, and high energy density”, and higher risk of dementia. Substituting over-processed foods with unprocessed or minimally processed foods can reduce this risk [15].

**Figure 2:**
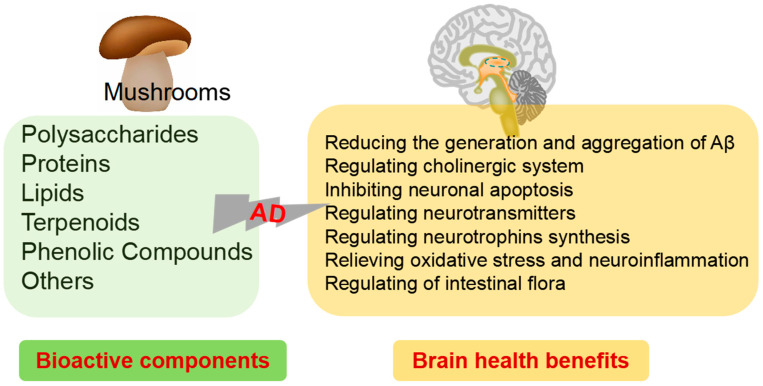
Bioactive components of mushrooms and brain health benefits.

Diet is one of the most important weapons we have in the battle against AD, which is designed to prevent the development of cognitive problems as well as to slow down further decline in symptoms in patients who have already been diagnosed with AD. The study of food-derived active ingredients is a promising area of research in the field of AD and provides a potential new avenue for the prevention and treatment of this debilitating condition. The nutrition and health benefits of mushrooms has been recognized by more and more people. “One meat, one vegetable and one mushroom” is the most reasonable dietary structure for human beings as recommended by the FAO. To provide more information for their potential applications in medicine as well as in functional foods designed for the intervention of AD, this review summarizes the biochemical composition and biological properties from mushrooms related with AD (Figure 2). Moreover, the underlying mechanisms for their neuroprotective activity are also highlighted.

## 2. Mushrooms against AD

There has been an explosion in research on nutritional interventions for preventing and treating AD, with promising results. Owing to their nutritional and medicinal values, mushrooms have been used for centuries. Mushrooms, a class of macroscopic fungi, plays an important role in the daily human diet due to their unique taste, umami flavor, and beneficial nutritional and medicinal properties. Mushrooms have a low fat content and are rich in nutrients, including high-quality proteins, dietary fibers, vitamins, minerals, and phenolic compounds [16,17]. There is a growing number of in vitro and in vivo trials describing a range of possible health benefits, including antioxidant, anti-inflammatory, anticancer, antimicrobial, antidiabetic, immunomodulatory, cardiovascular-protective, hepato-protective, geno-protective, and neuro-protective effects [18,19,20,21].

Recent studies have suggested that mushroom intake shows potential in preventing and alleviating cognitive impairment associated with AD. The relationship between mushroom intake and dementia incidence was studied in a group of elderly Japanese subjects aged older than 65 years. This cohort study showed that frequent consumption of mushrooms was significantly associated with a decreased risk of incident dementia and might have a preventive effect on the risk of dementia [22]. The research team of the National University of Singapore, School of Medicine found that the elderly consuming at least two standard mushrooms (about 300 g) per week were found to reduce the risk of mild cognitive impairment (MCI) by 50% [23]. A study on the correlation between mushroom intake and cognitive ability in elderly Americans suggested that higher mushroom intake might reduce the risk of cognitive decline in older adults [24]. Consumption of culinary medicinal mushrooms might significantly lower the risk of age associated neurodegenerative disorders, such as AD [25]. Extensive research on culinary and medicinal mushrooms has demonstrated their neuroprotective effects, which include the ability to prevent neuronal death and regulate both NDs and neurotrauma [26]. This work comprises six preclinical and three clinical studies that effectively illustrate the potential benefits of Hericium erinaceus extracts and bioactive compounds in ameliorating cognitive function and behavioral deficits in animal models of AD. Remarkably, the clinical trials yielded similar results to the preclinical studies [27]. Mushrooms and their bioactive molecules provide significant neuroprotective effects and play a vital role in preventing the onset and advancement of NDs [28]. We investigated the protective effects of mushroom polysaccharides, proteins, lipids, terpenoids, phenolic, and other biologically active compounds on AD.

### 2.1. Polysaccharides

**Figure 3:**
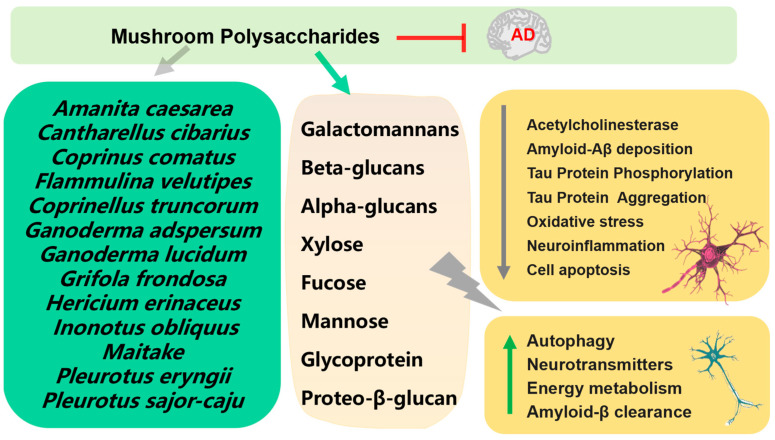
Mechanism of prevention and treatment of AD by mushroom polysaccharides.

Numerous mushroom polysaccharides have exhibited neuroprotective effects in different neurodegenerative models in vivo and in vitro (Figure 3) [29]. One of the ways to treat AD is to control the function of the neurotransmitter acetylcholine in the brain by inhibiting acetylcholinesterase (AChE). Polysaccharides extracts, which mostly contain β-glucans, from *Coprinus comatus* and *Coprinellus truncorum* exerted AChE inhibitory activity [30]. Two new galactomannans I and II isolated from *Rhizopogon luteolus* and *Ganoderma adspersum* mushrooms were tested for their antioxidant and anticholinesterase activity; the results showed that galactomannan II showed significantly strong anticholinesterase activity [31]. Proteo-β-glucan from *Maitake* ameliorated cognitive impairments by enhancing microglial Aβ clearance in a APP/PS1 mouse model [32]. Polysaccharides from *Pleurotus eryngii* showed the protective effect on Aβ-induced neurotoxicity in rat pheochromocytoma cells (PC12) and aging rats [33]. Polysaccharides purified from *Hericium erinaceus*, composed of two high molecular weight polysaccharides (molecular weights—1.1 × 10^5^ Da and 1.7 × 10^5^ Da) showed antioxidant and neuroprotective effects on Aβ-induced neurotoxicity in PC12 [34]. Orally administrated polysaccharide isolated from *Amanita caesarea* to APP/PS1 to mice for 6 weeks significantly improved cognition behavior through regulation of oxidative stress-mediated endoplasmic reticulum (ER) stress [35].

Polysaccharide fractions (CC2a, CC3) of *Cantharellus cibarius* had beneficial effects on neuron activity and neurite outgrowth under normal and different stress conditions. Additionally, both CC2a and CC3 fractions exhibited antioxidant ability and could effectively neutralize negative changes induced by glutamatergic system activators [36]. Polysaccharide PSP2-1, derived from *Pleurotus sajor-caju*, exhibited neuroprotective effects on mouse hippocampal neuronal cells (HT22) from hydrogen peroxide (H_2_O_2_)-induced oxidative damage and apoptosis via the MAPK signaling pathway. In addition, PSP2-1 could also improve the cognitive ability of aging mice induced by D-galactose [37]. Oral administration of *Grifola frondosa* polysaccharides to 20-month-old rats for 8 weeks could improve memory deficits via antioxidant action [38]. Huang and coworkers extracted water-soluble polysaccharides and alkaline-soluble polysaccharides from *Ganoderma lucidum* and evaluated their antioxidant and hepatoprotective effects on a mouse model of AD-induced acute liver damage using carbon tetrachloride [39]. Administration of *Ganoderma atrum* polysaccharide (PSG-1) showed a protective effect on the oxidative stress induced by D-galactose in mouse brain, and significantly reduced apoptosis in mouse brain in a dose-dependent manner, which attributed to its capacity to increase endogenous antioxidants activity and attenuate intracellular calcium accumulation [40].

Mycelium polysaccharides extracted from *Armillaria mellea* showed a protective effect in L-glutamic-acid-induced HT22 cell apoptosis and an aluminum trichloride (AlCl_3_) plus D-galactose-induced AD mouse model [41]. Polysaccharides from *Flammulina velutipes* by compatibilizing with ginsenosides exhibited cognitive-enhancing effect on D-galactose induced AD rats [42]. *Pleurotus ostreatus* polysaccharides was found to alleviate cognitive impairment in a D-galactose and AlCl_3_-induced rat model of AD [43]. A 3-h pre-treatment with *Amanita caesarea* polysaccharides (ACPS) before L-glutamic acid co-exposure was observed to ameliorate the damage in HT22 cells through the activation of the nuclear transcription factor erythroid-2-related factor 2 (Nrf2) pathway. In the AD mouse model induced with D-galactose and AlCl_3_, an administration of 2.5 or 5 mg/kg ACPS for 42 days showed improvement in cognitive impairment [44]. Polysaccharides purified from *Inonotus obliquus* (IOPS) showed a protective effect in L-glutamic acid exposed HT22 cells; orally administered IOPS (25 or 50 mg/kg once daily for 8 weeks) improved the memory and cognition impairment in APP/PS1 transgenic mice [45].

Mushroom-derived polysaccharides are susceptible to degradation by gut microbiota, serving as an energy source for certain bacterial groups that promote their growth and production of beneficial compounds, notably short-chain fatty acids (SCFAs), such as acetic, propionic, butyric, and valeric acid [46]. Qian et al. [47] investigated the impact and mechanisms of SCFAs on AD-related cognitive function, pathological features, and neuroinflammation [47]; SCFAs derived from gut microbiota can be used as potential therapeutic targets for AD.

### 2.2. Proteins

Mushrooms are a rich source of proteins; the protein content of mushrooms is far higher than that of wheat, rice, corn, and other food crops, as well as higher than that of various fruits and vegetables. Therefore, mushrooms are a good source of protein for vegetarians. Due to their richness in high-quality proteins, mushrooms are a promising source of bioactive peptides. At present, many bioactive peptides, such as antihypertensive, antioxidant, antimicrobial, anticancer, and other active peptides, have been discovered in various mushrooms [48]

Inflammation plays an essential role in various NDs, including AD [49]. This inflammatory reaction is supported by the activity of glial cells, such as astrocytes and microglia around the neurons [50]. Activation of glial cells is closely related to neuroinflammation. Novel selenium peptides obtained from selenium-enriched *Cordyceps militaris* showed protective effect in H_2_O_2_-injured PC12 and alleviated the cognitive impairment in lipopolysaccharide (LPS) injured mice through its antioxidative, anti-inflammatory, and regulating properties on gut microflora [51].

*Pleurotus geesteranus* protein hydrolysates, prepared using different enzymes (papain, alcalase, flavourzyme, pepsin, and pancreatin), showed that the alcalase hydrolysate exhibited superior in vitro antioxidant activity; at the same time, alcalase hydrolysate exhibited neuroprotective effects in H_2_O_2_-injured PC12 via reducing the accumulation of reactive oxygen species (ROS) in cells by stimulating the activity of antioxidant enzymes [52]. *Pleurotus geesteranus* hydrolysates with a higher abundance of hydrophobic amino acids obtained by simulated gastrointestinal digestion exhibited neuroprotective effects on H_2_O_2_-damaged PC12, possibly by reducing ROS production and enhancing the activity of the antioxidant enzyme system [53].

Ergothioneine (ET) is a natural sulfur-containing amino acid that cannot be synthesized by humans, but is rich in diets, especially mushrooms [54]. Plasma levels of ET decline with age; low ET levels are one of the risk factors that makes individuals susceptible to NDs, while supplementation through diets could be beneficial [55,56] The neuroprotective capabilities of ET in a range of in vitro and in vivo models have been reported [57]. In the 5 × FAD mouse model, longitudinal consumption of 50 mg/kg ET can reduce Aβ plaques, oxidative stress, restore glucose metabolism, and delay the progression of AD [58]. Aminothioneine, a hydrophilic amino acid extracted from golden oyster mushrooms, enhanced the expression of brain-derived neurotrophic factor (BDNF) mRNA in primary rat cortical neuron cultures through Ca^2+^ signal-mediated cAMP-response element-binding protein (CREB)-dependent transcription of neurons [59].

### 2.3. Lipids

The fat content of mushrooms usually ranges from 0.1 to 16.3%; although they are not the preferred source of lipids, they contain essential fatty acids, including linoleic, oleic, and linolenic acid as their major components [60]. Moreover, the levels of unsaturated fat acids in mushrooms are usually higher than that of saturated fatty acids [61].

Alpha linoleic acid was found to protect the mouse brain from Aβ-induced glial-cell-mediated neuroinflammation, avoiding neuronal cell loss and improvement of memory deficits in a Aβ-infused mouse model [62]. Two kinds of mushrooms from Anatolia were prepared with hexane and methanol after baking and non-baking and their extracts and major fatty acids were evaluated for the AChE and butyrylcholinesterase (BChE), generally known as the chief enzyme of AD. The results showed that the methanol extract of *Ramaria flava* had the highest activity in scavenging 1,1-diphenyl-2-picrylhydrazyl (DPPH), 2, 2′-azinobis(3-ethylbenzothiazoline-6-sulfonic acid) (ABTS) and BChE assays, but the nutritional concentration and biological activity of *Lactarius delicious* decreased after baking [63].

Activated microglia produce nitric oxide (NO) free radicals. The prolonged accumulation of substantial amounts of NO in the central nervous system (CNS) can result in neuroinflammation, which is associated with AD [64] Fatty acids, fatty acid esters, and sterols present in an ethyl acetate fraction of *Cordyceps militaris* reduced NO production in a mouse microglial cell line (BV2) via activation of Nrf2 and NF-κB pathways [65]. Reducing neuronal cell death is important for preventing and treating NDs. Dilinoleoyl-phosphatidylethanolamine (DLPE), a phosphatidylethanolamine bearing two linoleic acids from *Hericium erinaceum*, was found to protect mouse neuroblastoma (N2a) from ER stress-induced cell death by the protein kinase C (PKC) pathway [66].

A total of eight novel cerebrosides were identified as neuritogenic compounds from *Termitomyces albuminosus*, among which four were newly discovered cerebrosides, named termitomycesphins A–D. These unique cerebrosides were isolated from the ethanol extract of *Termitomyces albuminosus* and featured a distinctive C19 hydroxylated sphingosine base with middle branches. Cerebrosides A and C contained a C16 α-hydroxy fatty acid, exhibiting higher neuritogenic activity compared to cerebrosides B and D, which had a C18 α-hydroxy fatty acid [67]. Termitomycesphins E and F, two new cerebrosides hydroxylated near the middle of the long chain base (LCB), were isolated from *Termitomyces albuminosus*. They have been proved to induce neuronal differentiation in PC12, while the main cerebroside obtained from *Termitomyces albuminosus* had no activity on PC12 due to no hydroxylation near the middle of the LCB, indicating the importance of additional hydroxyl group on LCB [68] Termitomycesphins G and H, two recently discovered cerebrosides derived from *Termitomyces albuminosus*, showed neuritogenic activity on PC12. Termitomycesphin G, featuring a 16-carbon chain fatty acid, displayed superior neuritogenic activity compared to Termitomycesphin H, which contained an 18-carbon chain fatty acid. This suggests that the length of the fatty acid chain plays a critical role in the neuritogenic activity [69]. These studies have shown that the structure of novel cerebrosides may be crucial in neuritogenic activity.

Linoleic acid and linolenic acid serve as the precursor of arachidonic acid (ARA) and docosahexaenoic acid (DHA), which are beneficial to brain health. Polyunsaturated fatty acids (PUFAs) and their derivatives play essential roles in various brain processes, including neurotransmission, cell survival, and neuroinflammation, thereby influencing mood and cognition. Diet and drugs targeting PUFAs may lead to novel therapeutic approaches for the prevention and treatment of brain disorders [70].

### 2.4. Terpenoids

**Figure 4:**
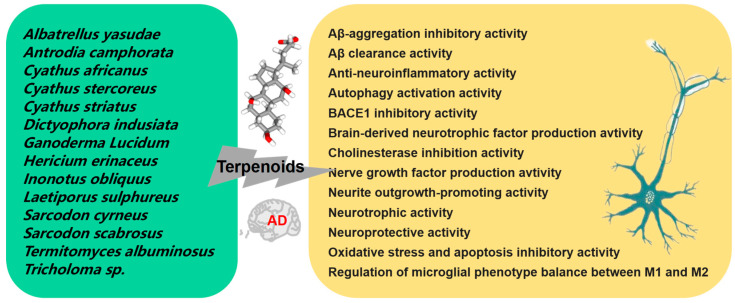
Mechanism of the prevention and treatment of AD by mushroom terpenoids.

Terpenoids, a prominent class of secondary metabolites found in mushrooms, are characterized by units of five-carbon atoms isoprene. Terpenes form the core of these compounds, and the addition of functional groups results in the formation of various terpenoids. This group comprises volatile unsaturated hydrocarbons classified as monoterpenoids, diterpenoids, sesquiterpenoids, and triterpenoids [71]. Figure 4 summarizes the currently reported mushroom species that produce active terpenoids and their potential therapeutic mechanisms for AD.

Four new selinane-type sesquiterpenoids and two known sesquiterpenoids were obtained from the fermentation broth of *Termitomyces albuminosus*; Epi-guaidiol A showed significant anti-AChE activity in a dose-dependent manner [72]. Four new meroterpenoids, namely scutigeric acid, albatrelactone methyl ester, albatrelactone, and 10′,11′-dihydroxygrifolic acid, as well as two known compounds, grifolin and grifolic acid, were extracted from the methanol extract of *Albatrellus yasudae*. Thioflavin T detection showed that four new meroterpenoids and methyl ester of scutigeric acid possessed inhibitory activity against Aβ aggregation, while all the six compounds exhibited inhibitory activities on beta-site APP-cleaving enzyme (BACE1) [73]. Ten compounds, comprising three novel meroterpenoids and seven known compounds, were obtained from the trichloro-methane extract of *Albatrellus yasudae*. Among these, six compounds showed the potential of Aβ-aggregation inhibition activity [74]. Cyathane diterpenoid sarcodonin A isolated from *Sarcodon scabrosus* showed anti-neuroinflammatory activity in LPS activated microglia, which could be mediated by MAPK/NF-κB pathway reversed LPS-induced M1 polarization in microglia cells [75]. A new lanostane triterpenoid 2α-HI obtained from *Inonotus obliquus (Fr.) Pilat* possessed significantly neuroprotective capacity on neuroblastoma cell line (SH-SY5Y) against H_2_O_2_ stimulated oxidative stress and apoptosis by activating the Nrf2 and BDNF/TrkB/ERK/CREB pathways. Additionally, the neuroprotective effect of 2α-HI in zebrafish has also been preliminarily verified [76].

Ganoderic acid A (GAA) is a highly oxygenated tetracyclic triterpenoid, serving as the main active component of *Ganoderma lucidum*. In a D-galactose induced mouse model, GAA administration (20 mg/kg) for 60 days alleviated neuroinflammation by regulating the Th17/Tregs axis [77]. GAA also facilitated Aβ clearance by promoting autophagy through the Axl receptor tyrosine kinase (Axl)/P21 activating kinases1 (Pak1) pathway in BV2 cells. Moreover, GAA administration (100 mg/kg) for 16 days attenuated cognitive deficits in an AD mouse model with intracerebroventricular injection of aggregated Aβ_42_ [78]. Methyl ganoderate A acetonide and n-butyl ganoderate H, together with 16 known compounds from *Ganoderma lucidum*, were found to owe the ability of anti-AChE activity. Additionally, only lucidadiol and lucidenic acid N exhibited anti-BChE activity [79]. Shen and colleagues explored the effects of peptidyl arginine deiminase type IV (PADI4) and GAA on Aβ_25–35_ treated HT22 cells; the results showed that PADI4 mediated autophagy and participated in the role of GAA in delaying Alzheimer’s cells aging through the protein kinase B (Akt)/mammalian target of rapamycin (mTOR) pathway [80]. Ganoresinoid A, isolated from the fruiting bodies of *Ganoderma resinaceum*, significantly inhibited NO, interleukin-6 (IL-6), interleukin-1β (IL-1β), and tumor necrosis factor-α (TNF-α) levels in LPS activated BV-2. Furthermore, ganoresinoid A notably reduced LPS induced apoptosis by reducing mitochondrial membrane potential and ROS. Additionally, ganoresinoid A exhibited antioxidant effects in H_2_O_2_-induced SH-SY5Y cells [81]. In another study, sixteen compounds were isolated from the fruiting body of *Ganoderma leucocontextum*, including three new lanostane triterpenes and thirteen known compounds. Out of them, two compounds showed a protective effect on H_2_O_2_ induced damage of PC12 and exhibited promoting neurite outgrowth at a concentration of 50–200 μM [82].

Accumulated evidence has demonstrated that the inflammation in brain is the main cause of NDs including AD. Deacetyl ganoderic acid F (DeGA F), a triterpenoid compound derived from *Ganoderma lucidum*, demonstrated inhibitory activity against the inflammatory response of BV-2 cells stimulated by LPS. In an in vivo study using zebra fish, DeGA F inhibited the production of NO in LPS-stimulated embryos. Additionally, DeGA F also suppressed the serum pro-inflammatory cytokines IL-6 and TNF-α levels and reduced inflammatory response in LPS-stimulated mouse model by suppressing activation of microglia and astrocyte [83] The ten new cyathane-type diterpenoids, as well as four known diterpenes, isolated from the liquid culture of the *Cyathus africanus*, have shown differential anti-neuroinflammatory activity, especially compounds cyathin I and allocyafrin B_4_, by inhibiting the expression and activity of cyclooxygenase-2 (COX-2) and nitric oxide synthase (iNOS) in LPS and Aβ_1–42_-treated BV-2 [84]. Eight new highly polyoxygenated cyathane diterpenoids and three known congeners were isolated from the solid culture of *Cyathus africanus*. All of these 11 compounds exhibited differential neurotrophic activity via nerve growth factor (NGF)-induced neurite outgrowth in PC-12, while only allocyathin B_2_ displayed anti-neuroinflammatory activity by suppressing the production of NO in LPS-stimulated BV-2 cells [85]. Seven undescribed lanostane-type triterpenoids, namely inonotusol H-N isolated from the fruiting bodies of *Inonotus obliquus*, displayed inhibitory activity on the production of NO in LPS activated BV-2. In particular, inonotusol I and L showed the most effective inhibition on the production of iNOS and NO [86].

Neurotrophins, including NGF and BDNF, hold a crucial role in in the CNS. Many diterpenoids and triterpenoids isolated from mushrooms have been proved to have the activity of promoting neurite growth. Dictyophorines A and B, isolated from *Dictyophora indusiata*, have demonstrated their ability to promote the synthesis of NGF in astrocyte [87]. Two novel cyathane diterpenoids (erinacines Z1 and Z2) and six known diterpenoids were isolated from the submerged cultures of *Hericium erinaceus* and *Hericium flagellum*. Compounds erinacine A, erinacine B, CJ14.258, and erinacines Z1 significantly enhanced the production of NGF or BDNF in astrocytes [88]. Three diterpenoids tricholomalides A-C, derived from the methanol extract of the fruiting body of *Tricholoma* sp., significantly induced the growth of neurites at a concentration of 100 µM in PC-12 [89]. Two new diterpene named scabronines K and L and four known analogues, namely sarcodonins G, A and M, and scabronine H, were isolated from the fruiting body of *Sarcodon scabrosus*. Among these compounds, only sarcodonins G and A at 25 mM exhibited significant neurite growth promoting activity in the presence of 20 ng/mL NGF after 24 h of treatment [90]. Two novel cyathane diterpenes, namely cyrneine C and D, as well as previously isolated cyrneine A and B and glaucopine C, were isolated from *Sarcodon cyrneus*. Among these compounds, cyrneine B induced the strongest NGF gene expression in human astrocytoma cell line (1321N1); cyrneines A and B as well as glaucopine C induced neurite outgrowth in PC12 to a lesser extent [91]. Twelve triterpenoids were obtained from both the fruiting bodies of *Laetiporus sulphureus* and the mycelial culture of *Antrodia* sp. MUCL 56049. Several of them can effectively stimulate the expression of neurotrophin (NGF and BDNF) on 1321N1 and also enhanced the neurite outgrowth of PC-12 induced by NGF [92].

Erinacine A pretreatment exhibited a preventive effect on LPS-stimulated iNOS expression and NO production in BV-2 cells, and TNF-α expression in CTX TNA2 astrocyte cells. Additionally, in differentiated N2a cells treated with LPS-activated BV-2 conditioned medium, erinacine A pretreatment significantly increased cell viability and tyrosine hydroxylase expression, while inhibiting c-Jun N-Terminal kinase (JNK) and NF-κB phosphorylation [93]. Rascher and colleagues revealed that cyathane diterpenoid erinacine C induced the expression of neurotrophin NGF and BDNF in glial cells. Moreover, their study elucidated the potential downstream signal cascade of NGF-mediated differentiation in neural-like PC12 cells [94]. Four known labdane diterpenoids were purified from the fruiting body of *Antrodia camphorata*. Compounds 19-hydroxylabda-8(17), 12-didehydroandrographolide, 13-dien-16, 14-deoxy-11, and 15-olide showed protective effect against Aβ-damaged primary cultures of neonatal cortical neurons [95].

A review compiled the available information on the neural health properties of *Hericium erinaceus* mycelia, which are abundant in erinacines, a group of cyathin diterpenoids. Preclinical studies have indicated that incorporating mycelia rich in erinacines into the daily diet can improve AD symptoms [96]. However, there are also some exceptions; the separation of diterpenoids from mushroom fruiting bodies has an inhibitory effect on neurite outgrowth. From the fruiting body of *Sarcodon scabrosus*, researchers isolated a new cyathane diterpene named scabronine M along with 10 known compounds. In PC12, only scabronine M significantly inhibited dose-dependent NGF induced neurite outgrowth in the absence of cytotoxicity. This is the first report that this group of diterpene inhibited neurite growth in PC12 [97].

### 2.5. Phenolic Compounds

Phenolic compounds, aromatic hydroxylated substances with one or more aromatic rings and hydroxyl groups, are prevalent in mushrooms. These compounds encompass flavonoids, phenolic acids, hydroxybenzoic acids, hydroxycinnamic acids, lignans, tannins, stilbenes, and oxidized polyphenols, which exhibit antimicrobial, anticancer, and anti-inflammatory effects. Moreover, they can play a role in preventing various degenerative diseases, such as brain dysfunction, cardiovascular diseases, and aging [98,99].

Hispidin-derived polyphenols, heat-stable components derived from *Auricularia polytricha*, exhibited in vitro inhibitory activity of BACE1, responsible for releasing toxic amyloid peptide in the brain [100]. Hispidin from the mycelial cultures of *Phellinus linteus* exhibited inhibitory activity of BACE1. In addition, hispidin also inhibited prolyl endopeptidase, but it had a lower inhibitory effect on alpha-secretase and other serine proteases, such as chymotrypsin, trypsin, and elastase [101]. Eleven phenolic compounds were detected from methanol and hot water extracts from fruiting bodies of *Phellinus pini* with higher levels of inhibition of AChE and BChE. Additionally, their activities were demonstrated by inhibition of NO production and iNOS expression in LPS-induced RAW 264.7 macrophages, while the methanol extract exhibited neuroprotective effect against glutamate-induced cytotoxicity on PC-12 at 20 to 40 µg/mL [102]. The ethanol extract of *Stereum hirsutum* exhibited high anti-AChE, which might be attributed to the phenolic substances (high content of p-hydroxybenzoic acid) as well as possible detected amentoflavone [103]. Inonophenols B and C, obtained from *Inonotus hispidus*, exhibited the highest activity in promoting PC-12 neurite outgrowth at a concentration of 10 μM. Moreover, these phenolic derivatives effectively reduced NO generation in LPS-activated BV-2 cells [104].

Flavonoids play various neuroprotective roles within the brain [105,106]. Hu et al. studied the antioxidant activity and the neuroprotective effect of flavonoids isolated from *Flammulina velutipes* against H_2_O_2_-induced PC12 [107]. Subsequently, additional investigations were performed on the effects of six flavonoids extracted from *Flammulina velutipes*, namely arbutin, epicatechin, phillyrin, apigenin, kaempferol, and formononetin, concerning their impact on H_2_O_2_-induced oxidative damage in PC12 cells. The results indicated that all components, except apigenin, mediated the apoptosis of PC12 cells through the endogenous pathway [108].

Mushrooms are abundant in bioactive compounds with antioxidant properties, primarily attributed to phenolic compounds. Several studies investigated the antioxidant content of mushrooms from around the world [109,110,111,112,113]. Foods containing antioxidants can protect oneself from excessive free radicals in the body, thereby preventing oxidative damage related chronic diseases, such as AD.

### 2.6. Other Small Molecule Bioactive Compounds

**Figure 5:**
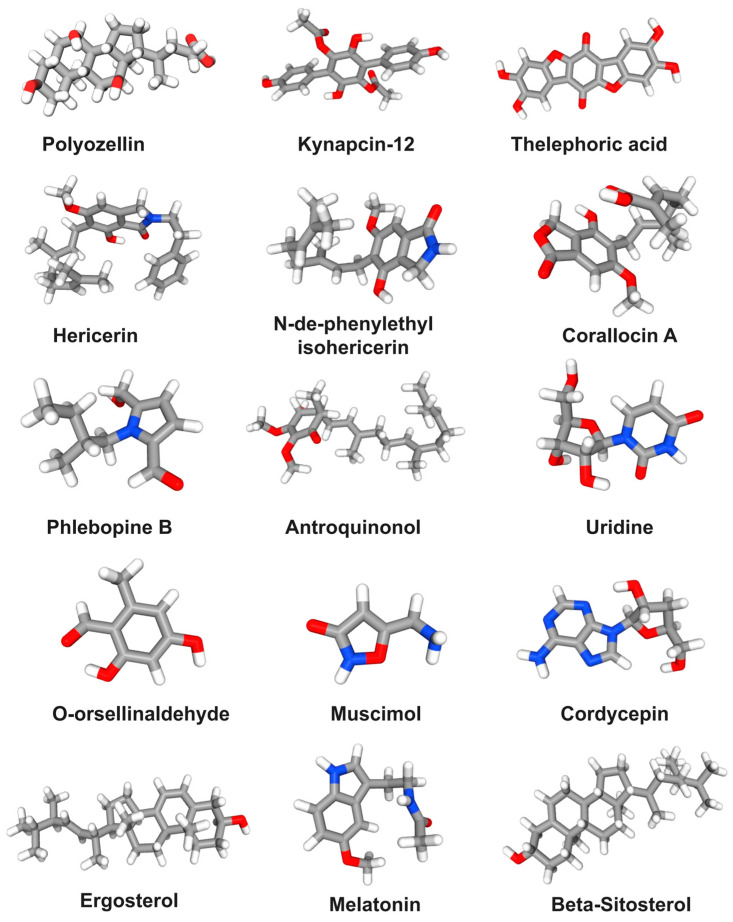
The structural formula of the main small molecule compounds in mushrooms for preventing and treating AD.

It is well accepted that mushrooms are a good source of proteins, fiber, and minerals. Furthermore, mushrooms also contain some bioactive compounds, which are essential for overall good health. Figure 5 lists the main small molecule compounds in mushrooms that have potential therapeutic effects on AD.

Polyozellin (25 μM) derived from *Polyozellus multiplex* alleviated HT22 death after 5 mM glutamate treatment for 12 h by inhibiting Ca^2+^ influx, intracellular ROS production, and lipid peroxidation. In addition, polyozellin also regulated the expression of Bid, Bcl-2, apoptosis-inducing factor, and phosphorylation of MAPK [114]. Four p-terphenyls, namely polyozellin, thelephoric acid, polyozellic acid, and kynapcin-12, were identified from the ethanol extract of *Polyozellus multiplex*. The results showed these compounds effectively inhibited the activity of BACE1. Polyozellin, thelephoric acid, and polyozellic acid reduced production of neurotoxicity Aβ_1–42_ in a dose-dependent manner in APPswe-N2a cells. Additionally, compounds thelephoric acid and polyozellic acid significantly restored cell viability when HT22 were subjected to 5 mM glutamate [115]. Pretreatment of p-terphenyl leucomentins (3 to 5 μM) from *Paxillus panuoides* for 1 h exhibited potent inhibitory effects against neurotoxicity of 50 μM H_2_O_2_ in mouse cortical cell culture [116]. Four compounds, namely hericerin, isohericerinol A, N-de-phenylethyl isohericerin, and corallocin A, were identified from *Hericium erinaceus*. Among them, isohericerinol A significantly increased the production of NGF in C6 glioma cells, followed by corallocin A and hericerin. In addition, the increased production of NGF by these compounds promoted the neurite outgrowth in N2a [117].

Dictyoquinazols A, B, and C, isolated from the methanol extract of *Dictyophora indusiata,* exhibited a dose-dependent protective effect on primary cultured mouse cortical neurons against glutamate and N-methyl-D-aspartate (NMDA)-induced excitotoxicity [118]. Seven pyrrole alkaloids were identified from the fruiting bodies of *Phlebopus portentosus*. A 2 h pretreatment of inotopyrrole B showed a significant neuroprotective effect against H_2_O_2_-stimulated neuronal-cell damage in SH-SY5Y [119]. Consumption of antroquinonol for two months, a ubiquinone derivative extracted from *Antrodia camphorata*, was found to reduce hippocampal Aβ levels and the degree of astrocyte proliferation and improved spatial learning and memory in a transgenic AD mouse model. These effects might be mediated by activating the Nrf2 pathway and reducing histone deacetylase 2 levels [120]. Moreover, 100 μM uridine from *Pleurotus giganteus* enhanced neurite outgrowth in N2a, which was due to increased phosphorylation of ERK, Akt, and mTOR. Moreover, mitogen extracellular signal-regulated kinase (MEK)/ERK and phosphatidyl inositol 3-kinase (PI3K)-Akt-mTOR further induced phosphorylation of CREB and expression of growth associated protein 43 [121]. O-orsellinaldehyde from *Grifola frondosa* strongly inhibited LPS-activated inflammation of primary microglia and astrocytes by reducing the formation of nitrite and downregulating the expression of iNOS and heme oxygenase 1 (HO-1). In addition, o-orsellinaldehyde inhibited NF-κB activation and effectively counteracted LPS-mediated p38 kinase and JNK phosphorylation (MAPK) in microglia cells; it also induced significant cellular immune regulation by repolarizing microglia into M2 anti-inflammatory phenotype [122]. A standardized extract from *Amanita muscaria* containing a large amount of muscimol revealed statistically significant neuroprotective effects on different neurotoxicity models of rat brain microsomes, mitochondria, synaptosomes, and SH-SY5Y. Moreover, it showed no inhibitory activity on human recombinant monoamine oxidase B [123]. In LPS-stimulated BV2, cordycepin extracted from *Cordyceps militaris* significantly inhibited the excessive production of NO, prostaglandin E_2_, and pro-inflammatory cytokines in a dose-dependent manner. Moreover, cordycepin blocked IkappaB-α proteins (IκB-α) degradation to suppress NF-κB activation and inhibited the phosphorylation of Akt, ERK-1/2, JNK, and p38 kinases [124].

Mushrooms are also a rich source of ergosterol. Ergosterol isolated from the ethanol extract of *Auricularia polytricha* was found to attenuate bisphenol A-induced BV2 inflammation through NF-κB signaling pathway [125]. In addition, they studied the protective effect of ergosterol prepared from *Auricularia polytricha* on TNF-α treated HT-22 damage and found that ergosterol was able to increase superoxide dismutase-1, the rapamycin-insensitive companion of mTOR, phospho-Akt, and ndusiat-glycogen synthase kinase-3β expression levels, and to suppress NMDA type subunit 2B gene transcription via overexpression of early growth response-1 [126]. Ergosterol can undergo UV treatment to be converted into Vitamin D2. Two-month-old AD transgenic mice were fed a Vitamin D2-deficient diet or a diet supplemented with 1 μg/kg of Vitamin D2 for 7 months. The AD transgenic mice fed with Vitamin D showed improved learning and memory abilities, a significant reduction in amyloid plaque load and glial fibrillary acidic protein, and an increase in interleukin-10 (IL-10) in the brain [127]. In another study, eighteen compounds were identified from the ethanol extract of *Dictyophora ndusiate*. The anti-inflammatory activity of seven isolated compounds were evaluated in BV-2 treated with LPS. Among them, a quinolone derivative demonstrated the most potent inhibitory effect on TNF-α expression. An ergosterol derivative exhibited the most effective activity in inhibiting IL-6 production. Another compound, namely 5α,6α-epoxy-7-sitosterol, showed anti-inflammatory effects through inhibiting NO and IL-1β generation and the expressions of iNOS and phosphorylated nuclear factor-kappa B inhibitor-α [128].

Mushrooms serve as a good dietary source of melatonin, which exhibits neuroprotective effects on CNS [129]. Shukla et al. [130] summarized the role of melatonin in cellular and animal models, as well as clinical interventions in AD patients, and explored the potential molecular mechanisms of melatonin action. Li et al. [131] investigated the cellular and molecular mechanisms of melatonin on various aspects related to AD, including Aβ generation, assembly, clearance, neurotoxicity, and circadian cycle disruption. Additionally, they summarized several clinical trials of melatonin for AD treatment. In an AD rat model, melatonin at a dose of 500 mg/kg improved spatial learning and memory impairment, restored synaptic plasticity, and reduced astrocyte proliferation through the Musashi1/Notch1/Hes1 signaling pathway following repeated intracerebroventricular administration of soluble Aβ_1–42_ [132]. It was reported that 10 mg/kg of melatonin administration restored the damaged memory in the hippocampus of aging mice and attenuated the decrease of α-secretase and inhibited the increase of β- and γ-secretases. Furthermore, melatonin weakened the upregulation of pNF-κB and the decrease of sirtuin 1 in the hippocampus of elderly mice [133]. In a 5 × FAD mouse model, treatment with 10 mg/kg melatonin improved the cognitive impairment through reversing the abnormal expression of protein in the lysosomal signaling pathway, mitochondrial energy metabolism, and pathological phagocytosis of microglia [134].

The diverse biological and physiological properties of bioactive components in mushrooms make them a natural dietary source for preventing and regulating AD. Herein, we have reviewed the preventive and therapeutic bioactive components related to various hypotheses of AD in mushrooms. The action mechanisms presented here include reducing the generation and aggregation of Aβ, regulating cholinergic system, inhibiting neuronal apoptosis, regulating neurotransmitters, regulating neurotrophins synthesis, relieving oxidative stress and neuroinflammation, and regulating of intestinal flora.

## 3. Conclusions

There is increasing evidence that certain lifestyle modifications, such as healthy diets, exercise, cognitive stimulation, etc., can help to reduce the risk of developing AD or slow its progression. Diets can be an effective tool for supporting and even improving cognition. Foods influence the brain in different ways; some nutrients affect the brain directly because they are capable of crossing the BBB, which acts as a gatekeeper to keep harmful chemicals out and allowing essential substances to come in. Foods also stimulate the release of certain chemicals, such as hormones and neurotransmitters, that influence brain function. Mushrooms may be a promising functional food for preventing AD. Mushrooms have many bioactive compounds that have the potential to regulate AD. These findings are encouraging; however, a substantial amount of research is still needed to study their optimal dose, limitations, bioavailability, the differences between chemical forms, and their possible interactions with other dietary components. Interactions between different components in mushrooms may produce antagonistic or synergistic effects to manage AD, but there are a limited number of studies on this; further deep research is needed to explore this. However, there is currently a lack of large-scale scientific investigations and clinical retrospective data analysis to confirm the positive effects of adding mushrooms to diets for the prevention and treatment of AD. Additionally, many of these compounds have not been well studied in clinical trials, and more rigorous studies are necessary to fully evaluate their safety and validate its overall efficacy in human beings. We envision increased clinical data supporting the efficacy of food therapy in AD prevention. Furthermore, we eagerly await the discovery and clinical application of novel bioactive compounds derived from mushrooms, offering promising prospects for enhancing AD prevention and treatment, and ultimately improving public health.

## Data Availability

The data used to support the findings of this study can be made available by the corresponding author upon request.
